# Supplementary material for: Product-stabilized filamentation by human glutamine synthetase allosterically tunes metabolic activity
Source: eLife. 2026 Oct 12;14:RP108336. doi: 10.7554/eLife.108336 (PMC13657631; doi:10.7554/eLife.108336)
Supplement: Supplementary file 2 [file elife-108336-supp1.docx]

### **Supplementary file 1. Cryo-EM data collection, refinement and validation statistics.**

Data collection parameters, reconstruction statistics, and model refinement and validation metrics for the apo filament (PDB 9OTQ, EMD-70845), turnover filament (PDB 9OTM, EMD-70841), turnover decamer (PDB 9OTO, EMD-70843), ATP filament (PDB 9OTN, EMD-70842) and R298A turnover decamer (PDB 9OTP, EMD-70844). Models were validated with Q-score as a plugin function for ChimeraX.

| **Structure** | **Apo Filament** | **Turnover Filament** | **Turnover Decamer** | **ATP Filament** | **R298A decamer** |
| --- | --- | --- | --- | --- | --- |
| **Ligands** | Mg(II) | ADP, Mg(II) | ADP, Mg(II) | ATP, Mg(II) | ADP, Mg(II) |
| **PDB code** | 9OTQ | 9OTM | 9OTO | 9OTN | 9OTP |
| **EMDB code** | EMD-70845 | EMD-70841 | EMD-70843 | EMD-70842 | EMD-70844 |
| **Magnification** | 105,000 | 106,000 | 106,000 | 105,000 | 134,000 |
| **Voltage (kV)** | 300 | 300 | 300 | 300 | 300 |
| **Electron fluence (e⁻/Å²)** | 60 | 45 | 40 | 64.2 | 40 |
| **Defocus range (μm)** | 0.8-1.8 | 0.6-2.0 | 0.6-2.0 | 0.8-1.8 | 0.6-2.0 |
| **Pixel size (data collection) (Å)** | 0.4215 | 0.82 | 0.86 | 0.4215 | 0.664 |
| **Pixel size (reconstruction) (Å)** | 0.843 | 0.82 | 0.86 | 0.843 | 0.664 |
| **Symmetry imposed** | D5 | D5 | C1 | D5 | C5 |
| **Particle images (no.)** | 112,643 | 468,883 | 2,043,084 | 310,500 | 619,435 |
| **Resolution (0.143 FSC) (Å)** | 2.27 | 2.19 | 2.03 | 2.11 | 1.95 |
| **R.m.s. deviation bond lengths (Å)** | 0.004 | 0.004 | 0.004 | 0.004 | 0.004 |
| **R.m.s. deviation bond angles (°)** | 0.942 | 0.925 | 1.018 | 0.933 | 1.026 |
| **MolProbity score** | 1.02 | 1.13 | 1.42 | 0.96 | 1.26 |
| **Clashscore** | 2.36 | 3.42 | 2.95 | 1.92 | 3.11 |
| **C-beta outliers (%)** | 0.00 | 0.00 | 0.00 | 0.00 | 0.00 |
| **Rotamer outliers (%)** | 0.14 | 0.14 | 1.15 | 0.32 | 0.55 |
| **Ramachandran favored (%)** | 98 | 98 | 96 | 98 | 97 |
| **Ramachandran allowed (%)** | 2 | 2 | 4 | 2 | 3 |
| **Ramachandran outliers (%)** | 0 | 0 | 0 | 0 | 0 |
