# Supplementary material for: Product-stabilized filamentation by human glutamine synthetase allosterically tunes metabolic activity
Source: eLife. 2026 Oct 12;14:RP108336. doi: 10.7554/eLife.108336 (PMC13657631; doi:10.7554/eLife.108336)
Supplement: Supplementary file 3 [file elife-108336-supp2.docx]

### **Supplementary file 2. Ligand model statistics for the deposited structures.**

Cross-correlation (CC), Q-score and B-factor for every modelled ligand in the apo filament (PDB 9OTQ), ATP filament (PDB 9OTN), turnover decamer (PDB 9OTO), turnover filament (PDB 9OTM) and R298A turnover decamer (PDB 9OTP). Values are given as average ± s.d. across the ten chains, with maximum and minimum in parentheses. CC and Q-score were measured with models fit into sharpened maps in ChimeraX; B-factors were derived from Phenix and deposited with the structures.

| Structure | Ligand | CC  Average ± SD (Max, Min) | Qscore  Average ± SD (Max, Min) | Bfactor  Average ± SD (Max, Min) |
| --- | --- | --- | --- | --- |
| Apo Filament | Mg2+ | 0.52 ± 0.06 (0.60, 0.42) | 0.80 ± 0.02 (0.83, 0.77) | 94 ± 3 (99, 89) |
| ATP Filament | Mg2+ | 0.77 ± 0.07 (0.86, 0.53) | 0.88 ± 0.03 (0.93, 0.81) | 24 ± 7 (36, 10) |
| ATP Filament | ATP | 0.76 ± 0.03 (0.81, 0.71) | 0.80 ± 0.03 (0.84, 0.73) | 27 ± 9 (37, 18) |
| Turnover Decamer | ADP | 0.83 ± 0.08 (0.87, 0.78) | 0.80 ± 0.01 (0.81, 0.78) | 86 ± 4 (92, 76) |
| Turnover Decamer | Mg2+ | 0.71 ± 0.04 (0.79, 0.65) | 0.86 ± 0.05 (0.93, 0.75) | 74 ± 6 (85, 58) |
| Turnover Filament | ATP | 0.82 ± 0.02 (0.85, 0.79) | 0.69 ± 0.03 (0.71, 0.64) | 123 ± 16 (140, 105) |
| Turnover Filament | Mg2+ | 0.77 ± 0.04 (0.88, 0.60) | 0.83 ± 0.06 (0.91, 0.60) | 114 ± 22 (149, 74) |
| Turnover Filament | Gln | 0.71 ± 0.04 (0.75, 0.66) | 0.51 ± 0.08 (0.59, 0.40) | 96 ± 3 (100, 93) |
| Turnover R298A | Mg2+ | 0.67 ± 0.08 (0.76, 0.51) | 0.87 ± 0.03 (0.91, 0.79) | 71 ± 9 (89, 59) |
| Turnover R298A | ADP | 0.77 ± 0.01 (0.78, 0.76) | 0.71 ± 0.03 (0.76, 0.68) | 86 ± 2 (88, 84) |
