# Supplementary material for: Product-stabilized filamentation by human glutamine synthetase allosterically tunes metabolic activity
Source: eLife. 2026 Oct 12;14:RP108336. doi: 10.7554/eLife.108336 (PMC13657631; doi:10.7554/eLife.108336)
Supplement: Supplementary file 6 [file elife-108336-fig1-figsupp1-data2.zip › Figure1_BSG_Coomassie_Fig1E_top_and_Fig1S1D_left_labelled.pdf]

Figure 1—source data: full raw uncropped SDS-PAGE gel, Coomassie detection

Crosslinker: BSG (bis-sulfosuccinimidyl glutarate). Bands shown in Figure 1E (top panel) and Figure 1—figure supplement 1D (left panel).

Unedited scan. Labels and guides are drawn in the surrounding margin; no part of the gel image has been altered.

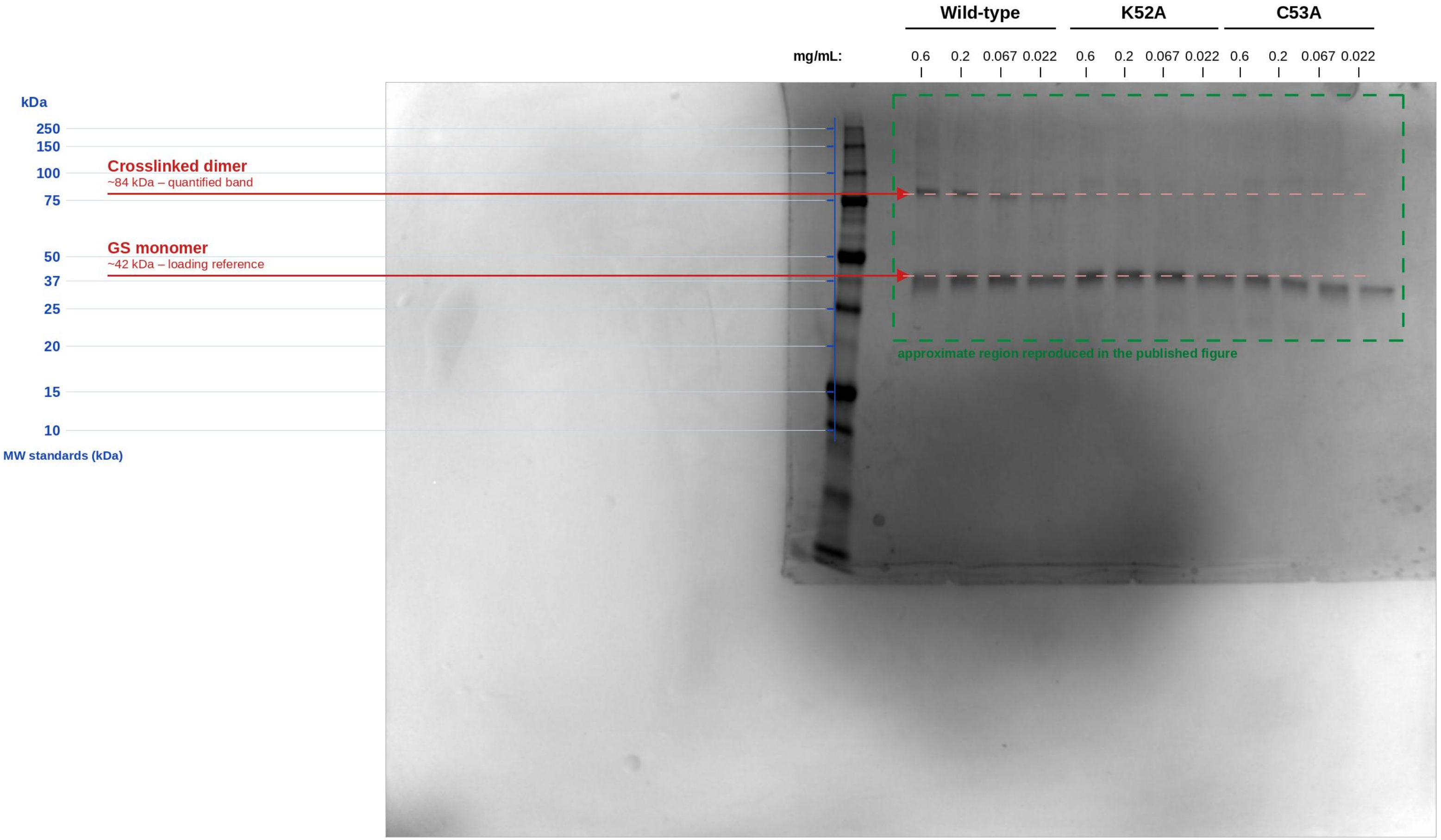

Lane order (left to right): MW ladder, then Wild-type, K52A and C53A GS, each at 0.6, 0.2, 0.067 and 0.022 mg/mL.

Crosslinked dimer accumulates only in wild-type GS; it is lost in the K52A and C53A filament-interface variants.

Red arrows: bands quantified in the figure. Green dashed box: region cropped for display. Blue ticks: MW standards.
