# Supplementary material for: Product-stabilized filamentation by human glutamine synthetase allosterically tunes metabolic activity
Source: eLife. 2026 Oct 12;14:RP108336. doi: 10.7554/eLife.108336 (PMC13657631; doi:10.7554/eLife.108336)
Supplement: Peer review history

# Editor report: eLife Assessment

**DOI:** 10.7554/eLife.108336.3.sa0

**Authors:** Luke Wiseman

**Affiliations:** Scripps Research Institute United States

**Keywords:** Solid, Useful

This manuscript employs cryo-EM, mutational analysis, and biochemical assays to explore the molecular basis by which glutamine promotes filamentation and regulates the activity of human glutamine synthetase (hGS) by stabilizing interactions between hGS decamers. The combined structural and biochemical data supporting the proposed mechanism are **solid**, although the evidence for a higher-order filamentous architecture and the precise assignment of the interface density would benefit from additional support. This work will be of particular interest and **useful** to groups interested in understanding the molecular basis of nutrient sensing, cellular metabolism, and structural regulation of enzyme activity.

# Referee report: Reviewer #1 (Public review)

**DOI:** 10.7554/eLife.108336.3.sa1

Summary:

The study is methodologically solid and introduces a compelling regulatory model. However, several mechanistic aspects and interpretations require clarification or additional experimental support to strengthen the conclusions.

Strengths:

(1) The manuscript presents a compelling structural and biochemical analysis of human glutamine synthetase, offering novel insights into product-induced filamentation.

(2) The combination of cryo-EM, mutational analysis, and molecular dynamics provides a multifaceted view of filament assembly and enzyme regulation.

(3) The contrast between human and *E. coli* GS filamentation mechanisms highlights a potentially unique mode of metabolic feedback in higher organisms.

Comment on revised version.

The authors have addressed all of my comments and concerns. The revisions have substantially improved the quality of the manuscript. I have no further questions or concerns.

# Referee report: Reviewer #2 (Public review)

**DOI:** 10.7554/eLife.108336.3.sa2

Major concern 1: The manuscript does not clearly establish a bona fide GS filament state.

The authors repeatedly refer to GS "filaments," but the data presented appear to support primarily a di-decameric assembly rather than a well-defined filamentous polymer.

A two-decamer reconstruction can define a putative inter-decamer interface, but it cannot by itself demonstrate propagation of a repeating filament geometry. To establish a bona fide filament, the authors should provide evidence for a reproducible one-dimensional assembly, such as at least three consecutive repeating units or equivalent quantitative evidence that the same inter-decamer transform propagates along an assembly axis.

In the current manuscript, many of the supporting 2D classifications appear to contain at most two adjacent GS decamers. This is particularly evident in the time-resolved cryo-EM datasets shown in Supplementary Figures 9-10, where I do not see convincing 2D classes corresponding to filaments. The same concern applies to other datasets, including Supplementary Figures 2, 6, and 11, where the apparent assemblies are primarily two-decamer particles.

Moreover, many of the selected "filament" classes show only one well-resolved GS decamer, while the neighboring decamer density is blurred. This suggests substantial variability in the relative position and/or orientation of adjacent decamers. Such heterogeneity is difficult to reconcile with a stable repeating filament geometry.

Therefore, the authors should explicitly define what they mean by "filament." If their evidence supports only a di-decameric or short oligomeric assembly, the terminology should be changed accordingly throughout the manuscript.

Symmetry concern

Given the low quality and heterogeneity of the 2D classifications for the putative "filament" classes, the use of D5 symmetry requires stronger justification. The current reconstructions primarily show the result after applying D5 symmetry to a two-decamer assembly. The authors should show reconstructions of the same particle sets processed under C1, C5, and D5 symmetry, and explain why D5 symmetry is justified.

This is particularly important because the claimed interface density and ligand interpretation are sensitive to symmetry averaging. Without showing how the reconstruction behaves under less restrictive symmetry assumptions, it is difficult to determine whether the final D5 map reflects a true biological assembly or a symmetry-imposed interpretation.

Filament abundance and physiological relevance

Even under the authors' broad classification criteria, the filament-like population appears to be a minor species. In some datasets, especially Supplementary Figure 10, the apparent filament fraction is very low, approximately 2-10%. This raises a major concern: if GS filaments are rare even under high-concentration cryo-EM conditions, are they expected to form to a meaningful extent under physiological conditions?

The authors propose a concentration-dependent assembly mechanism. If so, the relevance of GS filamentation in the lower-concentration cellular environment becomes even less clear. The authors should quantify filament abundance as a function of GS concentration and glutamine concentration, ideally under conditions closer to physiological ranges.

K52/C53 interface mutations

The authors use K52 and C53 as filament-interface residues, but the mechanistic contribution of these residues to filament assembly remains insufficiently explained. Why should K52A or C53A disrupt filament formation? Is the effect due to loss of a specific side-chain contact, altered local electrostatics, reduced crosslinker accessibility/reactivity, local structural destabilization, or nonspecific disruption of the interface?

The manuscript states that the interface is "concentration dependent and driven primarily by electrostatic interactions," but the data presented before that statement do not clearly establish this. The authors should explicitly identify the interacting electrostatic partners and provide structural or biochemical evidence supporting this interpretation.

Functional linkage between filamentation and kinetics is weak.

The authors should establish the oligomeric state of GS under the actual assay conditions. In particular, what is the filament fraction during the Figure 2F / Supplementary Figure 15 kinetic assays? Is the change in KM ammonia quantitatively correlated with filament abundance?

This is currently unclear. The direct comparison between decamer and 2-decamer fractions does not robustly show a functional difference, and the later glutamine-addition assays are interpreted as filament-mediated without directly demonstrating the filament fraction under the same assay conditions.

In Figure 2F, WT, K52A, and C53A already show different ammonia-dependent kinetic parameters in the absence of added glutamine. K52A and C53A appear to have lower basal kcat/KM ammonia and higher KM ammonia than WT even without glutamine. The authors should explain why these interface mutants already alter basal ammonia kinetics. Without such an explanation, K52A and C53A cannot be treated as clean controls that selectively disrupt glutamine-stabilized filamentation.

Major concern 2: The interface density is not convincingly assigned to glutamine.

The second foundational issue is the assignment of the interface density to glutamine. At present, the evidence is not sufficient to support the conclusion that glutamine is the ligand at this interface.

The local density at the interface appears weak and likely has lower local resolution than the reported global resolution. The current density could represent a low-occupancy or symmetry-averaged amino-acid-like density rather than a confidently assigned glutamine molecule.

Ligand pose and hydrogen bonding.

The proposed glutamine pose also requires more rigorous validation. The authors state that glutamine forms hydrogen bonds with interface residues, including K52, C53, and E55. These hydrogen bonds should be shown explicitly in a figure, with distances listed.

The proposed interaction involving C53 appears unusual and should be justified chemically and geometrically.

Glutamate has not been excluded.

The largest problem is that the authors do not adequately consider glutamate as an alternative ligand. They compare the density with phosphate and ATP/ADP, but this is not sufficient. Glutamate is present at high concentration during turnover, and it is chemically and structurally very similar to glutamine. Given the limited local density and possible orientational averaging, distinguishing glutamine from glutamate from the current cryo-EM density alone is not justified.

The authors should report or estimate the concentrations of glutamate and glutamine at the vitrification time point used for the high-resolution turnover-filament reconstruction. If glutamate is present at a much higher concentration than glutamine, the authors must explain why the interface density should be assigned to glutamine rather than glutamate.

The authors should fit both glutamine and glutamate into the interface density using the same validation criteria and compare the results. Stronger support would come from direct structural experiments, such as cryo-EM structures of GS incubated separately with glutamate and glutamine under controlled conditions.

Unless stronger evidence is provided, the claim that "glutamine binds to the filament interface" cannot be made.

Specific comments

Interface assembly statement:

"These data suggest that the formation of the interface is concentration dependent and driven primarily by electrostatic interactions."

What specific data support "concentration dependent" at this point in the manuscript? Which residues or chemical groups are proposed to form the electrostatic interactions? The authors should provide a more explicit explanation.

Line 149-150:

"In both scenarios, any signal is likely to be averaged out and experiments with symmetry expansion and focused classification did not yield any convincing density."

Please show these analyses. Negative results are important here because they bear directly on the reliability of the interface interpretation.

"Glutamine stabilizes larger GS filaments":

What does "larger" mean? Longer filaments, more decamers per filament, or larger diameter? The authors should define this quantitatively, preferably by reporting filament-length distributions or the number of decamers per assembly.

Filament classification:

The criteria used to classify particles or 2D classes as "filament" are not sufficiently clear. The authors should provide the full 2D classification results for each time-resolved dataset, including selected and discarded classes, particle numbers, and objective selection criteria. Some selected and discarded classes appear visually similar, especially in Supplementary Figures 9-10.

R298A decamer:

The R298A mutant is presented as a turnover-decamer structure, not a filament structure. The authors should clarify whether R298A forms filament-like particles under comparable turnover conditions. If R298A does not form filaments, this should be reported and explained. If filament-like particles were present but excluded during processing, the authors should provide their abundance and justify why only the decameric form was analyzed. This point matters because R298A is used to connect E305-loop disorder with the proposed filament-associated mechanism, although R298A is a loop-stabilization mutant rather than a filament-interface mutant.

Line 231-233:

"a reaction time that should yield a high concentration of product due to the higher enzyme concentration than previous experiments"

What is the estimated product concentration at vitrification? What concentration range qualifies as "high"? The authors should provide a quantitative estimate.

Glutamine hydrogen bonds:

The proposed hydrogen bonds linking glutamine to K52, C53, and E55 should be shown explicitly with atom identities and distances.

Glutamate comparison:

What is the glutamate concentration in the same sample? Given that glutamate is chemically similar to glutamine and likely present at high concentration, why is the interface density not glutamate? The authors should compare glutamine and glutamate fitting using the same validation criteria.

Line 248-253:

The speculation that apo filaments may arise from high GS concentration or residual glutamine should be moved to the Discussion. In the Results, this reads as an ad hoc explanation rather than a result directly supported by data.

Actual assay-state oligomeric distribution:

What is the filament fraction under the actual kinetic assay conditions? Is the KM ammonia change quantitatively correlated with filament abundance?

Figure 2F:

Why do WT, K52A, and C53A differ in basal ammonia-dependent activity even without added glutamine? The authors should explain whether these mutations alter intrinsic ammonia kinetics independent of filamentation.

Supplementary Figure 15 / Figure 2F:

Please clarify the relationship between Figure 2F and Supplementary Figure 15. The kinetic constants in Figure 2F appear to depend on global fitting of progress curves shown in Supplementary Figure 15. The authors should provide replicate-level raw progress curves, between-replicate variability, fitting residuals, and individual fitted parameters.

Supplementary Figure 19:

Supplementary Figure 19 should be presented consistently with Supplementary Figure 18, including the corresponding 2D classification results.

In summary, although the revised manuscript improves the presentation of cryo-EM map processing, the two foundational claims remain unresolved. The current data establish, at most, a di-decameric or filament-like GS assembly, but not a rigorously defined filamentous polymer. In addition, the interface density is not convincingly assigned to glutamine, particularly because glutamate has not been excluded as the most relevant alternative ligand. Since the proposed negative-feedback mechanism depends directly on these two points, the current evidence does not support the strength of the title, abstract, or mechanistic conclusions.

# Referee report: Reviewer #3 (Public review)

**DOI:** 10.7554/eLife.108336.3.sa3

In this manuscript, the authors propose a product-dependent negative-feedback mechanism of human glutamine synthetase, whereby the product glutamine facilitates filament formation, leading to reduced catalytic specificity for ammonia. Using time-resolved cryo-EM, the authors demonstrate filament formation under product-rich conditions. Multiple high-quality structures, including decameric and di-decameric assemblies, were resolved under different biochemical states and combined with MD simulations, revealing that the conformational space of the active site loop is critical for the GS catalysis. The study also includes extensive steady-state kinetic assays, supporting the view that glutamine regulates GS assembly and its catalytic activity. Overall, this is a detailed and comprehensive study. However, I would advise that a few points be addressed and clarified.

Comments on revised version.

The revision addresses several reviewer concerns: the authors add sharpened maps, ligand-density panels, symmetry expansion/focused classification, biochemical blank/substrate/TCEP controls, and E305-loop focused classification. The E305-loop part is stronger now: turnover decamer recovers partial E-flap density in few classes, while turnover filament does not.

My only remaining comment is that - as also the authors agree on the need to integrate density with biochemical data and that local resolution/averaging complicates modeling - I would advise softening the claim regarding glutamine from "glutamine binds" to "density consistent with glutamine/product-associated density". In general, it would be best to avoid overstating atomic certainty at the filament interface and the safest framing is the observed interface density is compatible with glutamine but not independently conclusive.

# Author comment: Author response

**DOI:** 10.7554/eLife.108336.3.sa4

**Authors:** Eric Greene, Richard Muniz, Hiroki Yamamura, Samuel E Hoff, Priyanka Bajaj, Markus CB Tecson, Cyrina Geluz, D John Lee, Erin M Thompson, Angelika Arada, Gyun Min Lee, Massimiliano Bonomi, Justin M Kollman, James S Fraser

**Affiliations:** Department of Bioengineering and Therapeutic Sciences, University of California San Francisco, San Francisco, CA San Francisco United States; Department of Biochemistry. University of Washington, Seattle WA Seattle United States; Department of Bioengineering and Therapeutic Sciences, University of California San Francisco, San Francisco, CA San Francisco United States; Institut Pasteur Paris France; Department of Bioengineering and Therapeutic Sciences, University of California San Francisco, San Francisco, CA San Francisco United States; San Francisco State University San Francisco United States; San Francisco State University San Francisco United States; Department of Bioengineering and Therapeutic Sciences, University of California San Francisco, San Francisco, CA San Francisco United States; Department of Bioengineering and Therapeutic Sciences, University of California San Francisco, San Francisco, CA San Francisco United States; Department of Bioengineering and Therapeutic Sciences, University of California San Francisco, San Francisco, CA San Francisco United States; Department of Biological Sciences, KAIST, Daejeon, 34141 Republic of Korea Daejeon Republic of Korea; Institut Pasteur Paris France; University of Washington seattle United States; Department of Bioengineering and Therapeutic Sciences, University of California San Francisco, San Francisco, CA 94158 San Francisco United States

The following is the authors’ response to the original reviews.

**Public Reviews:**

**Reviewer #1 (Public review):**

Summary:

The study is methodologically solid and introduces a compelling regulatory model. However, several mechanistic aspects and interpretations require clarification or additional experimental support to strengthen the conclusions.

Strengths:

(1) The manuscript presents a compelling structural and biochemical analysis of human glutamine synthetase, offering novel insights into product-induced filamentation.

(2) The combination of cryo-EM, mutational analysis, and molecular dynamics provides a multifaceted view of filament assembly and enzyme regulation.

(3) The contrast between human and *E. coli* GS filamentation mechanisms highlights a potentially unique mode of metabolic feedback in higher organisms.

Weaknesses:

(1) The mechanism underlying spontaneous di-decamer formation in the absence of glutamine is insufficiently explored and lacks quantitative biophysical validation.

(2) Claims of decamer-only behavior in mutants rely solely on negative-stain EM and are not supported by orthogonal solution-based methods.

We thank the reviewer for the summary and noting of the strengths. We agree that the evolutionary divergence of metabolic feedback in GS homologs is a fruitful avenue for future studies. With regard to the weaknesses, the di-decamer in the absence of glutamine only forms under high (higher than physiological) concentrations of enzyme. Our primary evidence for the mutant behavior was the lack of crosslinking (Figure 1E), with supplementary support from the negative stain. In the revised version we will soften the language to say “reduced” rather than “did not support” filament formation.

**Reviewer #2 (Public review):**

The authors set out to resolve the high-resolution structure of a glutamine synthetase (GS) decamer using cryo-EM, investigate glutamine binding at the decamer interface, and validate structural observations through biochemical assays of ATP hydrolysis linked to enzyme activity. Their work sits at the intersection of structural and functional biology, aiming to bridge atomic-level details with biological mechanisms - a goal with clear relevance to researchers studying enzyme catalysis and metabolic regulation.

Strengths and weaknesses of methods and results:

A key strength of the study lies in its use of cryo-EM, a technique well-suited for resolving large, dynamic macromolecular complexes like the GS decamer. The reported resolutions (down to 2.15 Å) initially suggest the potential for detailed structural insights, such as side-chain interactions and ligand density. However, several methodological limitations significantly undermine the reliability of the results:

(1) Cryo-EM data processing: The absence of critical details about B-factor sharpening - a standard step to enhance map interpretability - is a major concern. For high-resolution maps (<3 Å), sharpening is typically applied to resolve side-chain features, yet the submitted maps (e.g., those in Figures 1D, 2D, and supplementary figures) appear unprocessed, with density quality inconsistent with the claimed resolutions. This makes it difficult to evaluate whether observed features (e.g., glutamine binding) are genuine or artifacts of unsharpened data.

(2) Modeling and density consistency: The structural models, particularly for glutamine binding at the decamer interface, do not align with the reported resolution. The maps shown in Figure 2D and Supplementary Figure S7 lack sufficient density to confidently place glutamine or even surrounding residues, conflicting with claims of 2.15 Å resolution. Additionally, fitting a non-symmetric ligand (glutamine) into a symmetry-refined map requires justification, as symmetry constraints may distort ligand placement.

(3) Biochemical assay controls: While the enzyme activity assays aim to link structure to function, they lack essential controls (e.g., blank reactions without GS or substrates, substrate omission tests) to confirm that ATP hydrolysis is GS-dependent. The use of TCEP, a reducing agent, is also not paired with experiments to rule out unintended effects on the PK/LDH system, further limiting confidence in activity measurements.

Achievement of aims and support for conclusions:

The study falls short of convincingly achieving its goals. The claimed high-resolution structural details (e.g., side-chain densities, ligand binding) are not supported by the provided maps, which lack sharpening and show inconsistencies in density quality. Similarly, the biochemical data do not robustly validate the structural claims due to missing controls. As a result, the evidence is insufficient to confirm glutamine binding at the decamer interface or the functional relevance of the observed structural features.

Likely impact and utility:

If these methodological gaps are addressed, the work could make a meaningful contribution to the field. A well-resolved GS decamer structure would advance understanding of enzyme assembly and ligand recognition, while validated biochemical assays would strengthen the link between structure and function. Improved data processing and clearer reporting of validation steps would also make the structural data more reliable for the community, providing a resource for future studies on GS or related enzymes.

We disagree with the reviewer’s overall assessment.

With regard to sharpening and resolution: we examined sharpened maps and in a revised version will present additional supplementary figures showing these maps side by side. We note that the resolutions reported are global and that the most interesting features are, of course, in the periphery and subject to conformational and compositional heterogeneity. We will include supplementary figures of core side chain densities that are more like what are expected by the reviewer in the revision. With regard to modeling: the apo filament and turnover filament datasets were handled nearly identically. The additional density is therefore likely not artefactual to the symmetry operator - however, the lower resolution in this region noted by the reviewer is worthy of further exploration. The maps are public and we think this is the most plausible interpretation of the density, which we based primarily on the biochemical data and will include more speculation in the version.

With regard to the biochemical controls: we point the reviewer to Figure S1, which shows that omission of ammonia or glutamate in the wild-type (tagless) system removes any coupling of the reactions. We will perform the additional controls to publication quality in the revised version along with the TCEP control. We note that the reducing agent is present across all experiments, ruling out an effect on any specific result. The inclusion of TCEP is also very standard in other published uses of the Coupled ATPase assay (e.g. PMID: 31778111 and PMID: 32483380 by our first author)

Additional context:

Cryo-EM has transformed structural biology by enabling high-resolution analysis of large complexes, but its success hinges on rigorous data processing and validation steps that are critical to ensuring reproducibility. The challenges highlighted here are not unique to this study; they reflect broader issues in the field where incomplete reporting of methods can obscure the reliability of results. By addressing these points, the authors would not only strengthen their current work but also set a positive example for transparent and rigorous structural biology research.

All the data is public and the reviewer or anyone is free to reinterpret the maps and models - and we encourage that rather than just an interpretation of our static figures. In addition, we will upload the raw micrograph data for the apo filament and turnover filament datasets to EMPIAR prior to submitting the revision.

**Reviewer #3 (Public review):**

In this manuscript, the authors propose a product-dependent negative-feedback mechanism of human glutamine synthetase, whereby the product glutamine facilitates filament formation, leading to reduced catalytic specificity for ammonia. Using time-resolved cryo-EM, the authors demonstrate filament formation under product-rich conditions. Multiple high-quality structures, including decameric and di-decameric assemblies, were resolved under different biochemical states and combined with MD simulations, revealing that the conformational space of the active site loop is critical for the GS catalysis. The study also includes extensive steady-state kinetic assays, supporting the view that glutamine regulates GS assembly and its catalytic activity. Overall, this is a detailed and comprehensive study. However, I would advise that a few points be addressed and clarified.

(1) In Figure 2D and Supplementary Figure 7, the extra density observed between the two decamers does not appear to have the defining features of a glutamine. A less defined density may be expected given the nature of the complex, but even though mutagenesis assays were performed to support this assignment, none of these results constitutes direct and conclusive evidence for glutamine binding at this site. I would thus suggest showing the density maps at multiple contour thresholds to allow readers to also better evaluate the various small molecules under turnover conditions that cannot be well fitted based on this density map, helping to provide a more balanced interpretation of the results.

(2) On the same point regarding the density for the enzyme under turnover conditions, more details should be provided about the symmetry expansion and classification performed, and also show the approximate ratio of reconstructions that include this density. Did you try symmetry expansion followed by focused classification, especially on the interface region?

(3) The interface between the two decamers of the model needs to be double-checked and reassigned, especially for the residues surrounding the fitted glutamine. For example, the side chain of the Lys residue shown in the attached figure is most likely modeled incorrectly.

We thank the reviewer for the feedback. As noted above, we will include supplemental figures that show maps at multiple thresholds and sharpening schemes. We noted in the manuscript and above that our interpretation here is based on integrating biochemical evidence alongside the density and will make that even more clear in the revised manuscript. The filaments +/- the putative glutamine density were processed nearly identically, but we will attempt various schemes of focused classification/symmetry expansion in the revision as well. However, we point out that there is extensive averaging there that makes modeling a bit trickier than expected given the global resolution.

**Recommendations for the authors:**

**Reviewer #1 (Recommendations for the authors):**

Major Comments

(1) Limitation to Di-decamer Formation:

Could the authors clarify why hGS, when visualized by cryo-EM, predominantly forms di-decamers rather than extended filaments? Since glutamine bridges the two decameric rings, one would expect this to promote further polymerization. It remains unclear why longer filaments are not observed under the conditions used. Moreover, data in Figure 1B-1D are not mixed with Gln, and the mechanism of the apo-form filament formation is not clearly discussed. If K52 and C53 are in charge of filamentation, it is supposed to form a long filament, not a stack of 2 decamers. The kinetics of wild type and mutations, including K52A and C53A, are different. This confused me as K52 and C53 don't participate in the reactions of glutamine synthesis. Does data reduce catalytic efficiency with K52A or C53A mutation suggest that di-decameric GS exhibits a greater catalytic turnover rate than the pure decameric GS?

We thank the reviewer for pointing this out and it was indeed filament length that was a point of curiosity during the study. Prior to preprint, we repeated the freezing conditions under identical turnover conditions and with high protein concentration as reported and indeed found much longer filaments - pointing to the capacity of the system to form larger complexes. However, we only captured a couple of ‘screening’ images and did not collect a full second dataset. Therefore, we hypothesize that filament length may be stochastic based on the subtle differences of individual grid vitrification based on the assumption that decamers within a filament can freely and quickly exchange. However, as shown in the time-resolved cryoEM experiment, the fraction of particles that are characterized as participating in a filament form (length-agnostic metric), does not appear to be subject to individual grid vitrification conditions but rather by experimental conditions (concentration of reaction-derived glutamine).

The filament formation in apo state was not further explored because the concentrations required to achieve filament formation in this case were supraphysiological.

We have edited the discussion to emphasize this point more clearly:

“While the enzyme concentrations to achieve robust filamentation in the absence of glutamine are much higher than observed in cells, the protein concentrations used in our time-resolved cryoEM experiments where filamentation is correlated with accumulation of glutamine are within the range of intracellular GS concentrations in *S. cerevisiae* (Engel et al. 2025) and human cell lines (Wiśniewski et al. 2014).”

Regarding the ability of apo-GS to form filaments - we identified that these residues were important based on their structural location at the decamer: decamer interface (Figure 1D; away from the active site as pointed out) and because their individual mutation to alanine attenuated the ability to form higher order filaments (Figure 1E). Therefore, these mutants were crucial controls in the steady-state kinetic experiments reported (Figure 2E). Here, we used exogenous glutamine to seed/stabilize filaments because we identified glutamine as serving this function and, crucially, because we did not observe glutamine occupancy in the active site under turnover conditions (which would suggest an orthosteric feedback inhibition mechanism; Supplementary Figure 10). Under these conditions, the wild-type, filament-competent protein displayed a ~3-fold *K_M, ammonia_* increase with glutamine addition compared to no glutamine, which when considered in the context of the greater E-305 flap conformational heterogeneity speaks to a model of allosteric feedback inhibition. Importantly, K52A and C53A show no difference in *K_M, ammonia_* between the glutamine and no glutamine condition, suggesting that attenuation of filament formation at this interface via mutation, eliminates the kinetic deficit. Therefore, K52A and C53A are not product-inhibited in the same manner as wild-type GS.

We have clarified the discussion to emphasize this result:

“Importantly, point mutations of the interfacial residues do not show a *K_M, ammonia_* defect in the presence of glutamine, indicative of the importance of the filament form for product feedback.”

(2) Origin of Di-decamer Formation in the Absence of Glutamine:

While the manuscript demonstrates glutamine-stabilized filamentation, the spontaneous formation of di-decamers under apo conditions is not mechanistically explained. The observation of 10-mer, 20-mer, and 40-mer species in Figure 1B should be validated against molecular weight standards or through SEC-MALS. The inference of higher-order oligomers based solely on migration is insufficient. Additional characterization (e.g., SEC-MALS, AUC, or mass photometry) would clarify whether these assemblies are biologically relevant or incidental.

We thank the reviewer for pointing out the low precision of preparatory size exclusion chromatography assignments of GS molecular weight filament depicted in Figure 1. We have included calibration standards and assignment in Supplementary Figure 1 and updated Figure 1 to include the ambiguity of these assignments in panel B. It is important to note that GS has historically been underestimated in size via these methods and was originally assigned as an octamer for which there was previous consensus (PMID: 10708854). The low concentration requirements of mass photometry preclude its use for this purpose and we are not in a position to do SEC-MALS or AUC for this. Hopefully, the negative stain, crosslinking, and cryo-EM results are sufficient to indicate that we have correlated signals with the correct species!

(3) Validation of Interface Mutants as Decamer-only Species:

K52A and C53A mutants are used to disrupt di-decamer formation and are shown by negative-stain EM to exist as decamers. While supportive, this is qualitative. The inclusion of quantitative biophysical data (e.g., SEC-MALS or mass photometry) would more convincingly demonstrate that these mutants do not transiently assemble into higher-order oligomers. Furthermore, molecular measurements describing the spatial relationship of interface residues - such as the distance between K52 and E55 or between C53 residues of opposing decamers - would aid interpretation. The use of the term "adjacent" (line 530) is vague and should be made more precise.

We thank the reviewer for the thoughtful comments. Beyond negative-stain EM we also performed a biochemical validation of the filament interface through bi-functional crosslinking based on the premise that the new filament interface, as defined by the apo-filament structure, presented new/unique pairs of nucleophilic amino acid R-groups in close proximity. We used Bis-sulfosuccinimidyl glutarate (BSG) or bis-maleimoethane (BMOE) to covalently link adjacent primary amines and sulfhydryls respectively (Figure 1E). This experiment defines two key principles of GS filament formation in the absence of glutamine:

(1) It is concentration dependent. In the wild-type case there is a protein-dependent increase on crosslinking efficiency for both crosslinkers.

(2) It is dependent on C53 and K52. Mutation of C53 or K52 significantly attenuated crosslinking efficiency.

To make sure that these results are more prominent, we have now included a table of these intersubunit distances between epsilon amine groups of lysines and gamma sulfhydroxyl of cysteines groups based on the apo-filament structure and labeled this as either participating in the filament interface or not. Furthermore, in line with multiple reviewers comments, we have updated Figure 1D to include a sharpened representation of the map that shows strong side chain density for the amino acid side chains to further support these reported side chain distance measurements.

We thank the reviewer for pointing out the low precision of the SEC chromatogram interpretation of Figure 1B and the figure has been amended to show filaments of variable length, instead of defined length. We also included a calibration curve to Supplemental Figure 1B and estimated molecular weights. While these estimates of size are lower than ground truth it is important to note that GS has historically displayed smaller than predicted molecular weights via size exclusion chromatography and analytical ultracentrifugation where initial characterization papers defined the oligomeric state as an octamer rather than decamer (PMID: 10708854). These studies and the present indicate potential adherence to resin and/or other factors about the shape of GS that lead to longer retention. Lastly, these SEC procedures were performed as a preparative step rather than for analytical purposes, so resolution was not the ultimate goal.

(4) Terminology: "Scarless" hGS:

The term "scarless human glutamine synthetase" is unconventional and potentially confusing. If it refers to the wild-type sequence lacking N- or C-terminal tags or mutations, I recommend using the term "native hGS" for clarity.

We usually reserve “native” for proteins isolated from the original species and not recombinantly expressed (as here). So we will leave the term scarless in the document.

(5) Helical Parameters of Filament Assembly:

The manuscript states a ~26{degree sign} rotation (clockwise or counterclockwise?) between decamers in the filament, yet does not describe how this value was derived. Given that hGS filaments form helices, this parameter could be assessed via helical reconstruction. Is it possible the actual helical twist is ~30{degree sign}, implying 12 stacked decamers per full turn? Please elaborate on how the rotational angle was determined.

Rotation was determined through inspection of the apo-filament cryoEM map in ChimeraX where an outline of a pentamer from one decameric unit was rotated with respect to the outline of a pentamer across the filament interface and the rotation was measured. Helical reconstruction was not pursued in this work owing to the typically short filaments observed in micrographs and the relative ease by which a 20-mer species could be selected via traditional 2D and 3D classification/reconstruction methods.

We have added to the methods the following to better illustrate this measurement:

“Decamer: Decamer rotation across the filament interface was determined through inspection of the apo-filament cryoEM map in ChimeraX where an outline of a pentamer from one decameric unit was rotated with respect to the outline of a pentamer across the filament interface and the rotation was depicted in Figure 1D.”

(6) Time-Resolved Cryo-EM and Filament Growth:

The use of time-resolved cryo-EM is innovative; however, the accessible timescales are relatively short. I suggest complementing this approach with techniques such as dynamic light scattering (DLS) or mass photometry, which allow extended real-time monitoring of filament assembly over longer durations (e.g., hours). These methods can also provide higher temporal resolution and particle size distributions.

We thank the reviewer for this suggestion and agree that understanding the kinetics of filament formation is critical. While DLS and mass photometry are excellent for monitoring assembly over hours, our data indicates that GS filament formation occurs on a much faster timescale.

As shown in Figure 2C, when we added ATP and Glutamine directly to GS and vitrified the sample after only 5 minutes, the majority of particles had already formed filaments, indicating that the interaction had reached saturation. This contrasts with our time-resolved experiment, where the kinetics of filament formation were likely rate-limited by the enzymatic generation of glutamine rather than the assembly process itself.

Consequently, we anticipate that filament assembly occurs on the order of seconds or less—a timescale we interpret as a necessary prerequisite for a rapid and effective cellular feedback mechanism. Therefore, we believe the current cryo-EM data accurately captures the biologically relevant window of assembly.

(7) Cryo-EM Symmetry Imposition and Loop Flexibility:

The use of D5 symmetry in cryo-EM reconstructions may obscure conformational heterogeneity in flexible elements, such as the E305 loop. Since the authors used MD simulations to characterize loop dynamics, it would strengthen the study to also perform symmetry expansion followed by non-uniform refinement and alignment-free 3D classification of individual subunits. This could provide experimental validation of the proposed conformational variability.

We agree with the reviewer that symmetry enforcement can mask conformational heterogeneity, particularly for flexible elements like the E305 loop (the E-flap). To address this, we followed the reviewer’s suggestion and performed symmetry expansion on both the turnover decamer and filament consensus maps. This was followed by focused, alignment-free 3D classification on the asymmetric unit containing the E-flap.

Our analysis revealed a clear distinction: while 4 out of 12 turnover decamer classes showed partial density for the E-flap (class 3, 7, 8, and 12)—consistent with the flexibility observed in our MD simulations—none of the turnover filament classes demonstrated similar density. To ensure a direct comparison, we utilized a C5-expanded turnover decamer map to maintain an identical asymmetric unit to the D5-expanded turnover filament map. We note here that the turnover decamer consensus volume is different from the deposited map for which no symmetry was applied.

Despite the different initial symmetries (D5 for filaments vs. C5 for decamers), we utilized a C5-expanded decamer map to maintain an identical asymmetric unit. For transparency, we have uploaded this C5-refined consensus map and all resulting 3D classification maps to Zenodo. We agree that the text is now strengthened given this result and we have added the following to the main text:

“The differential loop density between turnover-decamer and turnover-filament species was further supported by 3D classification of symmetry-expanded particles, which recovered partial E305-loop density in 4/12 turnover-decamer classes (C5; Supplemental Figure 18) compared to 0/12 classes for the turnover-filament (D5; Supplemental Figure 19).”

(8) Crosslinking Gel Analysis (Figure 1E):

The SDS-PAGE gels shown in Figure 1E have molecular weight ladders cropped. For proper interpretation, please include full ladders with size markers and labels. In addition, clarify whether the crosslinked samples were denatured in reducing buffer. Crosslinking efficiency and specificity using BMOE or BSG require verification under reducing conditions (e.g., DTT, β-mercaptoethanol, or TCEP) to confirm covalent linkage between decamers.

We have added in Figure 1E molecular weight markers estimates to aid in gel interpretation and have included the uncropped gels in Supplementary Figure 1D that contain the full MW ladder. The methods were clarified to indicate that reducing reagent was used in both the crosslinking reaction and all SDS-PAGE samples.

“Protein samples were diluted to concentrations noted in base buffer (60 mM HEPES pH 7.6, 50 mM NaCl, 50 mM KCl, 10 mM MgCl_2_, 0.1 mM TCEP) and, reacted with crosslinker to a final concentration of 0.5 mM for 10 mins at room temperature followed by quench in 5X SDS-PAGE sample buffer (225 mM Tris pH 6.8, 50% glycerol, 0.05 % SDS, 0.2 mg/mL bromophenol blue, 1M DTT) supplemented with 100 mM of either NH_4_Cl (to quench BSG reactions only) or DTT (to quench BMOE). Protein concentrations were normalized after quench prior to SDS-PAGE analysis.”

(9) Missing Reference for NADH-Coupled Assay:

Line 678-679 refers to an NADH-coupled assay described "previously" without citing a source. Please provide a proper reference to ensure reproducibility.

The original paper describing the implementation of a coupled-assay to measure ADP production from glutamine synthetase was written by Bennett Shapiro and Eric Stadtman in 1970 and has been included. We will note that the conditions of this assay have been much improved since this time with better buffers, commercially available reagents of combined lactate dehydrogenase and pyruvate kinase, and modern plate readers. We added the following reference:

“Shapiro, B.M. and Stadtman, E.R., 1970. [130] Glutamine synthetase (*Escherichia coli*). In Methods in enzymology (Vol. 17, pp. 910-922). Academic Press.”

(10) Unclear Description of the NADH Assay:

The stability of NADH is influenced by pH and light exposure. Please specify the pH range used in the assay and whether precautions (e.g., light shielding) were taken. NADH autoxidation at high pH or degradation at low pH could impact assay reliability and should be addressed in the Methods section.

For clarity and transparency the following text was added to the Methods section.

“Stocks of ATP, NADH, and phosphoenolpyruvate were made in base buffer (60 mM HEPES pH 7.6, 50 mM NaCl, 50 mM KCl, 10 mM MgCl_2_, 0.5 mM TCEP) and the pH was adjusted until it reached 7.5 on ice prior to aliquoting, flash freezing, and storage at -80°C in the dark. NADH was only exposed to light upon thawing and assay set-up and no appreciable change in absorbance of control experiments were noted.”

(11) Ligand Density in Figure 2 and Supplementary Figure 7:

The density attributed to glutamine, ADP, and phosphate appears broader than expected. Please include cross-correlation (CC) values, estimated occupancies, and Q-factors for ligand fitting. Varying the contour level to assess density consistency would clarify whether the observed volume represents multiple conformations, partial occupancy, or overfitting. A similar concern applies to the cysteine sidechain density.

We have updated Supplemental Figures to include:

(1) Globally refined map in comparison to locally refined map where both are sharpened per previous feedback.

(2) Ligand placement now also include Q-scores and CC values

We did not include multiple contour levels because these are included in the resolution representative Supplemental Figure and because alternative contours do not influence Q-scores. From this analysis it is apparent that phosphate and ADP are both worse fits to the density.

Moreover, we have now included Supplementary Table 2 that includes all ligand validation statistics for the reader to evaluate the range of B-factor, CC values, and Q-scores for all ligands in all models.

(12) Missing Ligand B-factors in Supplementary Table 1:

The ligand refinement statistics in Supplementary Table 1 are incomplete. Please include B-factors and occupancy values for all ligands.

We have updated the PDB depositions to include B-factors in .cif files that are now available. We have also included Supplementary Table 2 in the manuscript detailing the ligand statistics for all models including cross-correlation, Qscore, and Bfactor.

(13) Style and Formatting Issues: format consistently throughout.

(a) Kinetic Parameters: Please follow the IUPAC and IUBMB-recommended formatting:

kcat should be italic with subscript.

KM should be italic K with upright M.

Use lowercase s^-1^, not uppercase S^-1^.

Refer to:

IUBMB enzyme nomenclature guidelines https://iubmb.org/wp-content/uploads/2021/01/Current_IUBMB_recommendations_on_enzyme_nome nclature.pdf

IUPAC Green Book https://publications.iupac.org/books/gbook/green_book_2ed.pdf

We have corrected the abbreviations according to the reviewers recommendations.

(b) Inconsistent Terminology and Typography:

cryo-EM vs. cryoEM are used inconsistently - standardize throughout.

FSC 0.143 appears with and without subscript formatting-please unify.

Line 123: "X-ray" should be capitalized.

Line 266: CryoEM should cryoEM, lowercase "c"

Line 571: "100 μg ml^-1^"-use superscript minus; ensure consistency with "mg ml^-1^".

Lines 605, 606, 626, 628: MgCl_2_-ensure the _2_ is subscripted throughout.

Temperature units (lines 607, 630, 640): Write as "4 {degree sign}C" instead of "4C".

Microliters (lines 660, 681, 697): Replace "uL" with "μL".

Line 797: Use superscripts: K^+^, Cl^-^.

Line 862: CO_2_ should appear with subscript.

We have made all terminology and typography consistent throughout based on these suggestions.

**Reviewer #2 (Recommendations for the authors):**

To strengthen the manuscript and address the methodological and interpretational gaps identified, we recommend the following revisions and additions:

(1) Data processing and cryo-EM map quality

(a) B-factor sharpening: Reprocess all cryo-EM maps using standardized B-factor sharpening workflows (e.g., the autoSharpen tool in cryoSPARC or similar methods) to enhance side-chain and ligand density visibility.

We have updated main and supplementary figures to include sharpened maps. All maps were sharpened using the Autosharpen feature of Phenix, specifically, by half-maps. We have included in the methods section the following to reflect this change:

“Final cryo-EM maps were sharpened in Phenix using the Autosharpen feature by half-maps.”

(b) Document the specific parameters used (e.g., B-factor values, solvent content estimates) in the Methods section to improve transparency.

See above regarding the additions made to the methods section.

(c) Map replacement and reanalysis: Replace all figures and supplementary panels displaying raw (unsharpened) maps (e.g., Figures 1D, 2D, 3A/B, 4A, 5B, and Supplementary Figs. 2D, S7B, S10) with the newly sharpened versions. Reanalyze density features (e.g., glutamine binding sites, ATP triphosphate groups) using these revised maps and update results to reflect any changes in interpretation.

As requested, we have updated figures with sharpened maps and found our original analyses to hold. In particular, we have included multiple metrics of ligand model scoring in Supplementary Figure 7B including Q-score and CC. Additionally, we have included all ligand model statistics in Supplementary Table 2.

(2) Structural modeling and validation

(a) Ligand fitting justification: Provide high-resolution ({less than or equal to}3 Å) density slices or side-chain density close-ups (e.g., for phenylalanine rings or glutamine-binding regions) to validate claims of atomic-level detail. For non-symmetric ligands (e.g., glutamine) fitted into symmetry-refined maps, explicitly describe how symmetry constraints were adjusted or applied during fitting (e.g., local symmetry refinement, manual adjustment of ligand orientation) and include validation metrics (e.g., cross-correlation scores, density fit plots) to support the placement.

We have supplied 5 new supplementary figures to demonstrate the resolution of our sharpened cryo-EM maps (most notably Supplementary Figures 3, 4, 8, 12, 22 and panels in others) . Of particular note is the sharpened map features of R298A decamer under turnover conditions which demonstrates multiple instances of a ring density for aromatic residues.

See discussion above regarding the placement of glutamine in the interface density and updated handling of symmetry during refinement.

(b) Ligand density supplements: Include supplementary figures showing representative ligand-density fits (e.g., ATP, glutamine) with clear side-chain or functional group annotations, as is standard in structural biology publications.

In our revision we have included the following updated figures and figure panels demonstrating ligand density into sharpened maps:

Turnover Filament Glutamine Ligand: Figure 2D-E (updated representation) and Supplementary Figure 7 (new and updated representations).

Turnover Filament ATP and Mg(II): Supplementary Figure 10 (updated representation)

Turnover Decamer ADP and Mg(II): Supplementary Figure 5 (new figure panel)

Turnover R298A ADP and Mg(II): Supplementary Figure 14 (new figure panel)

(3) Biochemical assay rigor

(a) Control experiments: Perform and report the following controls to strengthen enzyme activity claims:

- A blank control (reaction mixture without GS, ammonia, or glutamate) to quantify background ATP hydrolysis.

- Substrate omission controls (reactions lacking ammonia or glutamate) to confirm that ATP hydrolysis depends on both substrates and GS catalysis.

- A TCEP effect control (compare ATP hydrolysis rates with and without TCEP) to rule out reducing agent interference with the PK/LDH coupled assay.

We have provided blank, substrate omission, and TCEP controls in Supplementary Figure 1. These results demonstrate negligible ATP hydrolysis without complete substrate inclusion and do not indicate any impact from TCEP inclusion.

(b) Direct activity validation: Consider supplementing the coupled assay with a more direct measure of GS activity (e.g., quantifying inorganic phosphate release via malachite green assay) to cross-validate results.

On the merits of the PK/LDH coupled assay being used for >55 years to measure steady-state activity of glutamine synthetases and that it is a robust assay as supported by the additional control experiments presented above in Supplementary Figure 1 we have elected not to pursue tedious cross-validation with a non-continuous assay and believe our interpretation of the enzyme kinetic results hold.

(4) Writing and presentation clarity

(a) Methods detail: Expand the Methods section to explicitly describe:

- Cryo-EM data processing steps, including B-factor sharpening parameters, map reconstruction workflows, and any post-processing (e.g., filtering, masking).

- Criteria used to validate ligand fitting (e.g., density threshold values, manual vs. automated docking).

See above the revisions made in response to critique from review #1 which we will briefly summarize here:

We have included in the methods section the following to reflect this change:

“Final cryo-EM maps were sharpened in Phenix using the Autosharpen feature by half-maps.”

Focused masks are represented in Figure 2E, Supplementary Figure 18, and Supplementary Figure 19. The details around focused mask utilization are included in the revised figure captions and the following was included in the Methods.

“Focused masks were generated in ChimeraX (v.1.7 and above). Focused refinement and 3D classification (3 Å filter resolution, PCA initialization) were performed in cryoSPARC. Strategy of class picking and refinement are noted in Supplementary Figures 18 and 19.”

Map reconstruction workflows are present in the relevant Supplementary Figures. No post-processing steps beyond map sharpening in Phenix were carried out. In general, human GS represents a straightforward protein to reconstruction via cryo-EM.

Ligand identification criteria was described throughout the results section. Supplementary Figure 7 was revised to show sharpened density for either globally refined or locally refined maps fit with all three products of the glutamine synthetase reaction (ADP, Pi, and glutamine) individually showing the best CC and Qscore for glutamine. Beyond Supplementary Figure 7 we also combined both biochemical experiments and cryoEM to make this ligand assignment supported by:

(1) Time-resolved cryo-EM experiments that show increasing filament particles over reaction time (Figure 2B and Supplementary Figures 9 and 10)

(2) Glutamine+ATP cryo-EM screening (Figure 2C) showing long filaments

(3) Supplementary Table 2 showing reasonable ligand statistics for glutamine

To clarify this in the Methods sections we include the following statement:

“Ligands were placed with ISOLDE (v1.7) and those with >0.5 Qscore and supporting biochemical and/or literature precedent were built.”

(b) Results framing: In the Results, clearly distinguish between observations supported by sharpened maps and preliminary/unvalidated features. Avoid over interpreting density in unprocessed maps (e.g., referring to "glutamine binding" in Figure 2D without noting current density limitations).

We have updated our discussion of Figure 2D (and now also Figure 2E) to include discussion of only sharpened maps and noted current density limitations to the interpretation.

(5) Data and material availability

(a) Ensure all supporting data are publicly accessible:

- Upload raw cryo-EM movies, particle stacks, and processed maps to the Electron Microscopy Data Bank (EMDB) with appropriate accession codes.

- Deposit final atomic models in the Protein Data Bank (PDB) and reference these accession codes in the manuscript.

We deposited maps and models with accession codes in advance of review. The PDB and EMDB codes are available in Supplementary Table 1.

Furthermore, for the focused maps and focused classifications that were generated during the review, and for the benefit of not cluttering the PDB/EMDB, we have included these more specific analyses in Zenodo: 10.5281/zenodo.20298855.

- Provide detailed protocols for biochemical assays (e.g., TCEP handling, enzyme purification) in the Methods or as supplementary information to enable reproducibility.

See updates above to reviewer #1

(b) By implementing these revisions, the manuscript will better align with eLife's standards for methodological rigor, transparency, and reproducibility, allowing readers to confidently evaluate the study's contributions to structural and functional biology.

We agree!

**Reviewer #3 (Recommendations for the authors):**

(1) In line 252, it would be helpful to show negative-stain EM images for each SEC peak, further probing whether any peaks correspond to partially aggregated, as this could affect the measured Kcat and Km.

We aren’t in a position to do this experiment. We routinely check for aggregation by noting Absorbance at 340nm for non-specific scattering indicative of aggregation and observed no evidence of aggregation in our fractions.

(2) In Supplementary Figure 6, many of the classes in the "Selected Filament Classes" inset appear to be averages of closely spaced particles, which may bias the calculation and should be excluded. In the "Selected Decamer Classes", I would suggest removing the top-view particle classes, as these particles not only have significantly different ice penetration rates, but are also more difficult to distinguish in 2D classification.

We agree and have provided an additional, more strenuous cutoff, analysis of the tr-cryo-EM data wherein only classes that show clearly aligned decamers are included and all top views are omitted (Additional Supplementary Figure 6). We are happy to say that even with the more strenuous cutoffs that our main conclusions that filaments increase with forward reaction time holds.

(3) In line 445, "Figure 5A" should be corrected to "Figure 5B".

We thank the reviewer for pointing this out and have made the correction.
